# Activation of the Ion Channel TRPV4 Induces Epithelial to Mesenchymal Transition in Breast Cancer Cells

**DOI:** 10.3390/ijms21249417

**Published:** 2020-12-10

**Authors:** Iman Azimi, Mélanie Robitaille, Kaela Armitage, Choon Leng So, Michael J. G. Milevskiy, Korinne Northwood, Huai Fang Lim, Erik W. Thompson, Sarah J. Roberts-Thomson, Gregory R. Monteith

**Affiliations:** 1 School of Pharmacy and Pharmacology, College of Health and Medicine, University of Tasmania, Hobart, TAS 7005, Australia; iman.azimi@utas.edu.au; 2 School of Pharmacy, The University of Queensland, Brisbane, QLD 4102, Australia; m.robitaille@uq.edu.au (M.R.); kaela.armitage@gmail.com (K.A.); c.so@uq.net.au (C.L.S.); huaifang92@gmail.com (H.F.L.); sarahrt@uq.edu.au (S.J.R.-T.); 3 ACRF Cancer Biology and Stem Cells Division, The Walter and Eliza Hall Institute of Medical Research, Parkville, VIC 3052, Australia; milevskiy.m@wehi.edu.au; 4 School of Chemistry and Molecular Biosciences, The University of Queensland, St Lucia, QLD 4067, Australia; k.northwood@uq.edu.au; 5 UQ Centre for Clinical Research, The University of Queensland, Herston, QLD 4029, Australia; 6 Institute of Health and Biomedical Innovation and School of Biomedical Sciences, Queensland University of Technology (QUT), Brisbane, QLD 4102, Australia; e2.thompson@qut.edu.au; 7 Translational Research Institute, The University of Queensland, Brisbane, QLD 4102, Australia; 8 Department of Surgery, St. Vincent’s Hospital, University of Melbourne, Melbourne, VIC 3065, Australia; 9 Mater Research Institute, Translational Research Institute, The University of Queensland, Brisbane, QLD 4102, Australia

**Keywords:** TRPV4, EMT, calcium channel, breast cancer

## Abstract

Epithelial to mesenchymal transition (EMT) in cancer is important in therapeutic resistance and invasiveness. Calcium signaling is key to the induction of EMT in breast cancer cells. Although inhibition of specific calcium-permeable ion channels regulates the induction of a sub-set of EMT markers in breast cancer cells, it is still unclear if activation of a specific calcium channel can be a driver for the induction of EMT events. In this study, we exploited the availability of a selective pharmacological activator of the calcium-permeable ion channel TRPV4 to assess the direct role of calcium influx in EMT marker induction. Gene association studies revealed a link between TRPV4 and gene-ontologies associated with EMT and poorer relapse-free survival in lymph node-positive basal breast cancers. TRPV4 was an important component of the calcium influx phase induced in MDA-MB-468 breast cancer cells by the EMT inducer epidermal growth factor (EGF). Pharmacological activation of TRPV4 then drove the induction of a variety of EMT markers in breast cancer cells. These studies demonstrate that calcium influx through specific pathways appears to be sufficient to trigger EMT events.

## 1. Introduction

Epithelial to mesenchymal transition (EMT) is a fundamental process in embryogenesis and wound healing and is an important feature of cancer progression [1,2]. EMT in cancer cells represents a change in cellular phenotype often from a proliferative, epithelial-like state that is typically associated with a more rounded cellular morphology and higher levels of E-cadherin (E-Cad) to a more mesenchymal-like state, that is coupled with a spindle-like morphology, expression of proteins such as vimentin and the transcription factors Snail and Twist [1,3]. EMT in breast cancer cells is linked to increased cancer cell invasiveness and therapeutic resistance [1,3,4]. Several cellular signaling pathways are linked to EMT induction, including Ca^2+^ signaling [4,5,6].

Suppression of increases in cytosolic free Ca^2+^ ([Ca^2+^]_CYT_) reduces the induction of EMT genes normally induced by epidermal growth factor (EGF) and hypoxia in MDA-MB-468 breast cancer cells [7]. Ca^2+^ signal-sensitive EMT induction has now been demonstrated in a variety of cancer cell types, including those of the ovary [8], prostate [9], and liver [10]. However, it is clearly the nature of the calcium signal that is important in EMT induction since not all stimuli that elevate [Ca^2+^]_CYT_ induce EMT [7], and in many cases silencing of calcium-permeable ion channels such as TRPM7, TRPC1, and ORAI1 only inhibit the induction of some EMT markers [7,11,12]. Although there are widely available pharmacological activators for calcium-permeable ion channels such as the L-type voltage-gated Ca^2+^ channel activator BAYK8644 [13] and the TRPV1 activator capsaicin [14], the lack of readily available pharmacological activators for specific ion channels associated with EMT induction in cancer cells has made it difficult to fully define the role of the calcium signal in EMT. If Ca^2+^ influx via a specific pathway is sufficient to induce EMT, it would demonstrate that Ca^2+^ influx is not simply a required co-factor for established EMT inducing cellular signaling pathways but sufficient to elicit a response on its own. One calcium-permeable ion channel with indirect links to EMT and with a potent pharmacological activator is TRPV4 [15,16].

TRPV4 is a cation permeable ion channel with roles in osmotic and temperature sensing that is activated by a variety of endogenous factors (including arachidonic acid metabolites) and is a therapeutic target for several conditions, including pulmonary edema [17]. In breast cancer, TRPV4 levels are elevated in basal-like breast cancers compared with other molecular breast cancer subtypes [18,19]. Silencing of TRPV4 reduces migration and invasiveness in mouse 4T07 mammary cancer cells in vitro and suppresses the metastasis of 4T1 breast cancer cells in vivo [18]. The links between TRPV4 and EMT in breast cancer have so far been indirect and include the positive correlation between an “EMT score” and TRPV4 gene expression in human breast cancer samples [18], and the ability of the non-specific TRPV4 activator 4α-PDD to reduce E-cadherin levels in mouse 4T07 mammary cancer cells [20]. TRPV4 is associated with some aspects of EMT induced by specific stimuli such as transforming growth factor β1 (TGFβ1) in keratinocytes [15]; however, the ability of TRPV4 activation or indeed any calcium-permeable ion channel to induce EMT is unknown. In these studies, we sought to explore the relationship between TRPV4 gene expression levels and breast cancer molecular subtypes, prognosis, and EMT. Furthermore, we exploited selective TRPV4 pharmacological inhibition and activation to define the role of TRPV4 in EMT induction and to determine if activation of a specific calcium-permeable ion channel is sufficient to induce EMT in breast cancer cells.

## 2. Results

### 2.1. TRPV4 Expression Associates with Tumor Dissemination

**Figure 1:**
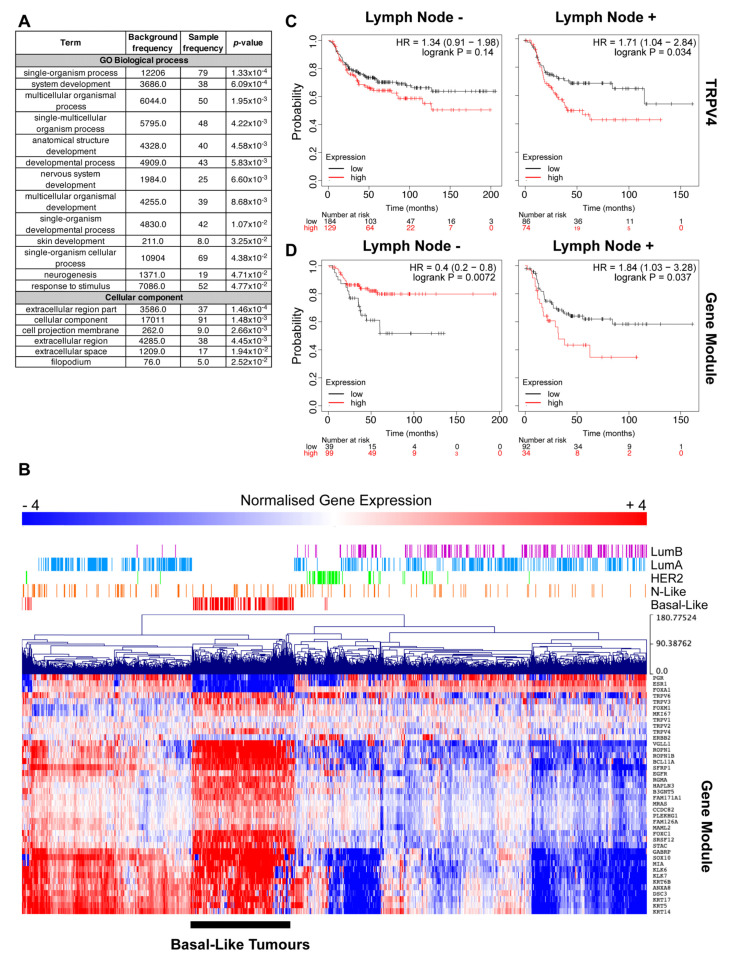
TRPV4 expression associates with tumor dissemination. (**A**) Gene ontology analysis of the Top 30 interconnected genes from the gene network module (Red Module) that TRPV4 is associated with and reveals links to biological processes and cellular components typical of developmental processes. (**B**) Hierarchical clustering of normalized gene expression from the Cancer Genome Atlas (TCGA) data focused on known markers of disease progression in breast cancer, including those that regulate epithelial to mesenchymal transition (EMT), TRPV channels, and the weighted gene co-expression network analysis (WGCNA) module. Expression values are mean-centered log2 normalized RNA-seq by expectation maximization (RSEM) values from breast cancer patients in the TCGA cohort. Basal-like tumors are noted. (**C**,**D**) Stratification of relapse-free survival (RFS) by TRPV4 and Red Module gene expression, respectively, in patients with basal-like breast cancer, separated by lymph node status. Patient numbers, *p*-value, and hazard ratios are indicated within the figure.

TRPV4 expression is elevated in basal-like breast tumors compared to other molecular subtypes [18,19]. We have previously demonstrated that TRPV4 expression is also associated with a breast cancer gene cluster (referred to as Red Module) that is strongly associated with EGFR, a gene often involved in the EMT process [19]. Basal-like breast tumors are highly heterogeneous, so to further explore the role of TRPV4 in this cancer type, we interrogated the Red Module by analyzing the gene-ontology of the top 100 most interconnected genes (Table S1) within this cluster (Figure 1A). This analysis revealed processes and cellular components involved in broad and general developmental processes. Gene expression of the Red Module across breast tumors revealed elevated expression in basal-like tumors, as previously identified [19]; however, there were some genes within this cluster that appeared to have heterogeneous expression within basal-like tumors (Figure 1B). Additionally, the Red Module was highly elevated in a subset of normal-like and luminal A tumors.

To further explore this heterogeneity in basal-like tumors, we stratified the relapse-free survival of patients with basal breast cancers and grouped them based on their lymph node (LN) status. This analysis revealed that high TRPV4 expression is a significant marker for patients with LN+ basal breast tumors, but not for patients with LN- disease (Figure 1C). Exploring the Red Module with these same parameters demonstrates that this module stratifies both LN- and LN+ disease, but intriguingly in LN- patients, high levels of this gene cluster are a predictor of better outcomes (Figure 1D); in contrast, high levels predict poor outcome in LN+ patients, similar to high TRPV4 expression (Figure 1D).

### 2.2. Regulation of Ca^2+^ Influx and Vimentin Protein Expression by TRPV4

**Figure 2:**
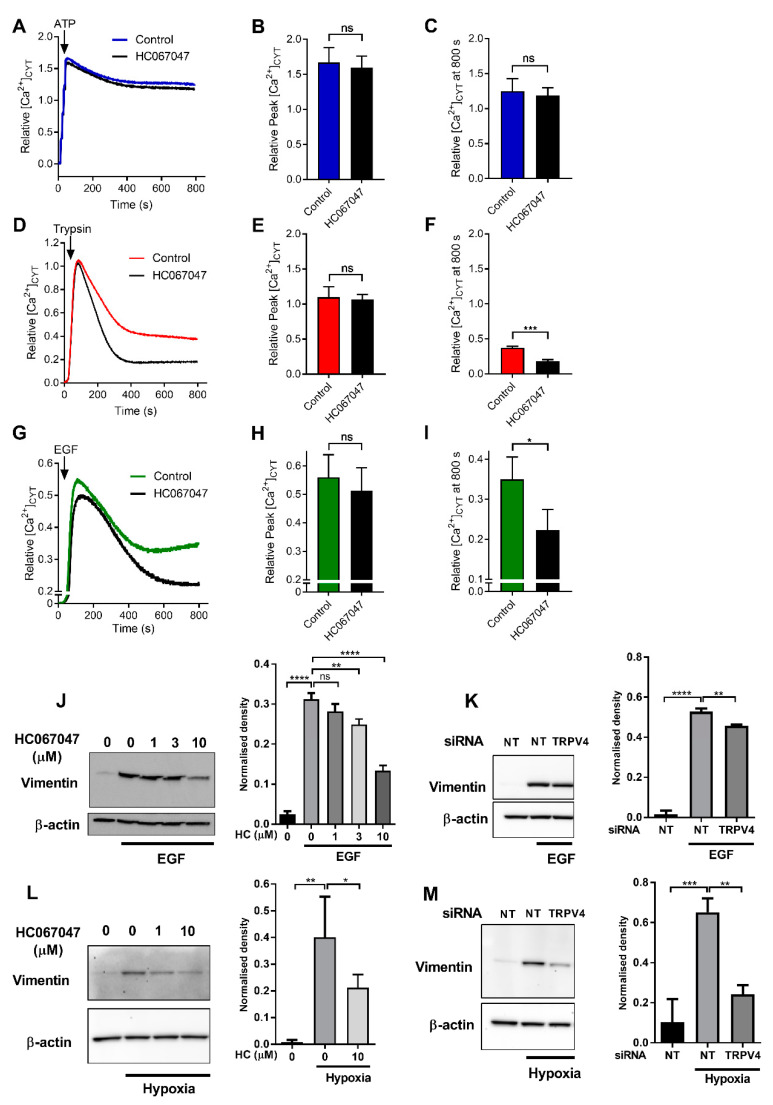
Regulation of Ca^2+^ influx and vimentin protein expression by TRPV4 in MDA-MB-468 cells. Average [Ca^2+^]_CYT_ transients with 100 µM ATP (**A**), 10 nM trypsin (**D**) and 50 ng/mL EGF addition (**G**) with or without HC067047 treatment (10 μM, 15 min pre-incubation and maintained throughout experiment). (**B**,**E**,**H**) Quantification of relative maximum peak corresponding to Ca^2+^ release from the ER and (**C**,**F**,**I**) quantification of relative [Ca^2+^]_CYT_ at 800 s corresponding to Ca^2+^ influx. Pooled data from three independent experiments, mean ± SD, ns = not significant, * *p* < 0.05, *** *p* < 0.001. (unpaired *t*-test). Representative western blot (left) and densitometric analysis of three independent experiments (right) of the effect of pharmacological inhibition of TRPV4 using HC067047 (HC) or TRPV4 siRNA-mediated silencing on (**J**,**K**) EGF-mediated (50 ng/mL for 24 h) and (**L**,**M**) hypoxia-mediated (1% O_2_ for 48 h) induction of vimentin protein expression. ns = not significant, * *p* < 0.05, ** *p* < 0.01, *** *p* < 0.001, **** *p* < 0.0001, one-way ANOVA, with Dunnett’s multiple comparisons, treatment groups compared to the second bar (EGF/hypoxia-induced control groups), *n* = 3, mean ± SD. NT= non-targeting.

The analysis described above indicated an association between TRPV4 and gene-ontologies associated with EMT, such as those linked to development. Given the indirect association between TRPV4 expression and EMT in breast cancer [15,18,20] and the importance of Ca^2+^ signaling in EMT [7], we sought to assess the possible role of TRPV4 in EMT induction. Our detailed assessment began with defining the contribution of TRPV4 to the sustained phase of Ca^2+^ influx induced by a variety of activators in EMT-inducible MDA-MB-468 breast cancer cells. Purinergic receptor activation produced a sustained increase in [Ca^2+^]_CYT_ (Figure 2A), which previous studies have shown to be sensitive to inhibitors of store-operated Ca^2+^ entry mediated by Orai1 Ca^2+^ channels [21]. Neither the initial increase in [Ca^2+^]_CYT_ elicited by ATP (Figure 2B) nor the sustained Ca^2+^ influx phase (Figure 2C) was sensitive to TRPV4 inhibitor HC067047. Assessment of [Ca^2+^]_CYT_ induced by low concentration of trypsin activating protease-activated receptor 2 (PAR2) (Figure 2D) showed that the sustained Ca^2+^ influx following PAR2 activation in MDA-MB-468 was in part mediated by TRPV4 (Figure 2E,F). Likewise, sustained Ca^2+^ influx induced by EGF (Figure 2G) was attenuated by TRPV4 inhibition (Figure 2H,I).

The ability of TRPV4 pharmacological inhibition to reduce Ca^2+^ influx produced by EGF (Figure 2G–I) led us to assess the consequences of TRPV4 pharmacological inhibition and silencing on EGF-mediated induction of the EMT marker vimentin, which is sensitive to TRPM7 inhibition in the same model [7]. HC067047 produced a significant and concentration-dependent inhibition of EGF-induced vimentin protein expression (Figure 2J). TRPV4 silencing (Figure S1) also significantly reduced the induction of vimentin expression by EGF (Figure 2K). TRPV4 pharmacological inhibition and silencing both reduced vimentin induction produced by hypoxia in this model (Figure 2L,M), another EMT inducer [7,22,23].

### 2.3. Promotion of EMT by TRPV4 Activation

**Figure 3:**
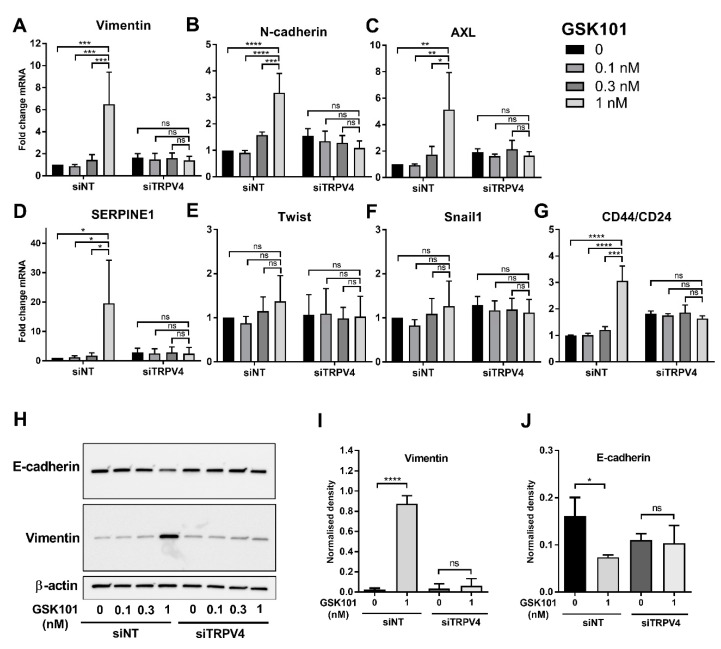
TRPV4 activation promotes EMT in MDA-MB-468 cells. (**A**–**G**) The effect of TRPV4 pharmacological activator, GSK1016790A (GSK101), on the mRNA expression of EMT markers in MDA-MB-468 cells treated with non-targeting siRNA (siNT) or TRPV4 siRNA (siTRPV4). ns = not significant, * *p* < 0.05, ** *p* < 0.01, *** *p* < 0.001, **** *p* < 0.0001 (two-way ANOVA, with Tukey’s multiple comparisons), *n* = 3, mean ± SD. Representative western blot (**H**) and densitometric analysis of three independent experiments (**I**,**J**) of the effect of GSK101 in TRPV4-silenced and control cells on the protein expression of vimentin and E-cadherin. ns=not significant, * *p* < 0.05, **** *p* < 0.0001 (one-way ANOVA, with Tukey’s multiple comparisons), *n* = 3, mean ± SD.

To determine whether TRPV4 mediated Ca^2+^ influx can drive EMT marker induction in this model, breast cancer cells were treated with low concentrations of the selective TRPV4 pharmacological activator GSK1016790A [16]. The concentrations of GSK1016790A were selected to be less than the EC_50_ for [Ca^2+^]_CYT_ increases in this breast cancer cell line (EC50 ~ 15 nM) and below the concentrations associated with cell death due to excessive sustained Ca^2+^ influx [19]. TRPV4 activation with GSK1016790A (1 nM) was sufficient to significantly increase mRNA levels of the EMT markers vimentin, N-cadherin, AXL, and SERPINE1 (Figure 3A–D). In all cases, this was abolished with TRPV4 silencing (Figure 3A–D). There was no significant induction of the EMT transcription factors Twist and Snail1 (Figure 3E,F); however, there was a significant increase in the stemness marker CD44/CD24 ratio with TRPV4 activation, and this was also eliminated with TRPV4 silencing (Figure 3G). TRPV4 pharmacological activation also induced EMT protein marker changes, as evident by the upregulation of vimentin protein (a mesenchymal marker) and downregulation of E-cadherin (an epithelial marker), which were also abolished by TRPV4 silencing (Figure 3H–J).

**Figure 4:**
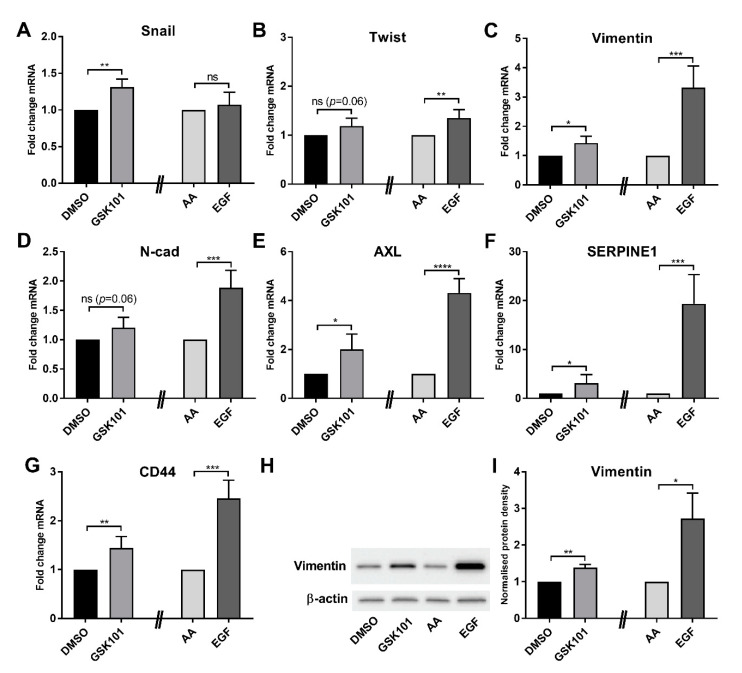
TRPV4 activation promotes EMT in PMC42LA cells. (**A**–**G**) The effect of TRPV4 pharmacological activator, GSK1016790A (GSK101), and EGF (10 ng/mL) on the mRNA expression of EMT markers in PMC42LA cells. Representative western blot (**H**) and densitometric analysis of three independent experiments (**I**) of the effect of GSK101 and EGF in TRPV4-silenced and control cells on vimentin protein expression in PMC42LA cells. ns = not significant, * *p* < 0.05, ** *p* < 0.01, *** *p* < 0.001, **** *p* < 0.0001, unpaired *t*-test, GSK101 compared to the dimethyl sulfoxide (DMSO) control, and EGF compared to its acetic acid (AA) control, *n* = 3, mean ± SD.

Assessment of the consequences of GSK1016790A treatment in another model of EMT in breast cancer cells supported the ability of TRPV4 activation to induce EMT marker expression. GSK1016790A produced a general trend in EMT marker mRNA levels in PMC42LA breast cancer cells with significant increases in Snail, vimentin, AXL, SERPINE1, and CD44 (Figure 4A–G). However, in all cases, the degree of increase was less than that induced by EGF and was less than the changes observed in MDA-MB-468 breast cancer cells. This may be reflective of less TRPV4 expression in PMC42LA breast cancer cells (Figure S2). Similar effects were seen with the induction of protein levels of the EMT marker vimentin (Figure 4H,I).

### 2.4. Regulation of Cell Motility by TRPV4

**Figure 5:**
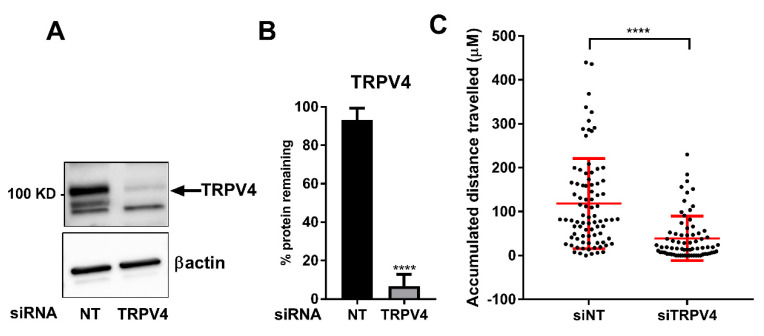
TRPV4 silencing reduces single-cell motility in MDA-MB-468 cells. Immunoblotting studies (**A**) and densitometry analysis (**B**) showing TRPV4 siRNA silencing, reducing TRPV4 protein levels by around 90%. **** *p* < 0.0001 compared with control (unpaired *t*-test), *n* = 3, mean ± SD. (**C**) A collagen-based model and individual cell tracking system were used to assess and quantify the migration of MDA-MB-468 breast cancer cells treated with non-targeting (NT) siRNA (total of 84 cells) and TRPV4 siRNA (total of 72 cells). In this model, the accumulative distance of individual cells migrated over the course of 12 h post-exposure to hypoxia (24 h) is assessed. **** *p* < 0.0001, (unpaired *t*-test). Graph represents the mean ± SD for three independent experiments.

One of the consequences of EMT can be a more migratory phenotype. To explore if TRPV4 may play a role in the migration of MDA-MB-468 breast cancer cells, the effects of TRPV4 silencing on single-cell migration was assessed. TRPV4 silencing (Figure 5A,B) significantly attenuated the accumulated distance traveled of single MDA-MB-468 breast cancer cells (Figure 5C).

## 3. Discussion

Basal-like breast tumors have elevated levels of TRPV4; however, the expression of TRPV4 within this breast cancer subtype is heterogeneous [18,19]. Our further assessment of basal-like breast cancers suggests that TRPV4 may contribute to or correlate with more invasive disease, given its association with poorer relapse-free survival in LN+ basal breast cancers but not LN-. Moreover, a gene cluster, which is associated with developmental processes and elevated expression in basal-like tumors [19], also stratifies patients with LN+ disease. These data suggest that TRPV4 might be part of a broader network of genes that are involved in the fundamental developmental process, such as EMT, that is utilized by tumors to progress invasion to the lymph nodes and beyond.

Increases in [Ca^2+^]_CYT_ can be achieved in breast cancer cells through the release of calcium from internal stores or the influx of Ca^2+^ ions across the plasma membrane. In some cases, the influx of Ca^2+^ is triggered by the release of Ca^2+^ from intracellular Ca^2+^ stores and influx through Orai1 channels. However, other channels can also contribute to Ca^2+^ influx after cell activation. For example, TRPV4 mediated Ca^2+^ influx occurs in a variety of cell types after stimulation of plasmalemmal receptors. Indeed, sustained Ca^2+^ influx induced by PAR2 activation is partly mediated via TRPV4, and this may be important in inflammatory pathways [24]. Our assessment of Ca^2+^ influx induced by a variety of stimuli demonstrated the differential contribution of TRPV4 to sustained Ca^2+^ influx induced by the purinergic receptor activator ATP, the PAR2 activator trypsin, and EGF in MDA-MB-468 breast cancer cells. ATP elicited a sustained increase in Ca^2+^ influx that was insensitive to TRPV4 inhibition. Although this could reflect an ability of ATP to induce Ca^2+^ influx by P2X channels, this is not the case given this sustained phase of Ca^2+^ influx is mediated by store-operated calcium influx in this model [21]. In contrast, the trypsin-induced Ca^2+^ influx was attenuated by TRPV4 pharmacological inhibition. Likewise, the sustained phase of Ca^2+^ increase induced by EGF was reduced by TRPV4 pharmacological inhibition, like the TRPV4-sensitive EGF activation pathways reported in renal and corneal epithelial cells [25,26]. In all cases, TRPV4 pharmacological inhibition had no effect on Ca^2+^ release from internal stores as reflected in similar initial peak [Ca^2+^]_CYT_ induced by all agents in the presence of HC067047.

A variety of EMT inducers are sensitive to intracellular Ca^2+^ chelation, and specific Ca^2+^ permeable ion channels can regulate EMT. However, in many cases, these Ca^2+^ permeable ion channels regulate only specific EMT markers and/or are specific to particular EMT inducers [7,11]. In contrast, our studies show that TRPV4 silencing and pharmacological inhibition attenuates vimentin protein induction induced by both EGF and hypoxia. The effect of the TRPV4 pharmacological inhibitor was greater than TRPV4 silencing on EGF-mediated effects. The TRPV4 pharmacological inhibitor at a maximal concentration will almost completely block TRPV4 channel activity, whereas siRNA mediated silencing will likely still leave around 10% of TRPV4 channels that could still be fully activated. The potential of TRPV4 to be of fundamental and direct importance in EMT induction was demonstrated through our assessment of direct TRPV4 activation with GSK1016790A. Direct TRPV4 activation increased levels of an array of mesenchymal markers and suppressed levels of the epithelial marker E-cadherin, all of which were abolished by TRPV4 silencing. These studies also indicate that at least some ion channels are not simply augmenters of EMT-inducing signals but appear to induce EMT in their own right. This may be important, given that some ion channels are activated by a variety of endogenous stimuli, such as is the case for TRPV4, where activation can be produced by mechanical deformation, changes in osmotic pressure, temperature, pH, and metabolites of arachidonic acid [27]. However, our studies do indicate that the ability and degree of ion channel-mediated EMT induction will be critically dependent on expression levels and appropriate cellular localization. MDA-MB-468 breast cancer cells where TRPV4 activation produced robust induction of EMT have high levels of TRPV4, in contrast to PMC42LA cells where TRPV4 levels are substantially lower and where TRPV4 activation produced far more modest and less diverse induction of EMT markers. The characterization of endogenous TRPV4 currents in MDA-MB-468 breast cancer cell lines is now warranted, given the potential role of such calcium influx in regulating cellular plasticity in this cell line

These studies highlight a new complexity and dimension to the targeting of some ion channels in breast cancer. Pronounced overexpression of TRPV4 in some breast cancers has been exploited in vitro and in vivo through pharmacological activation and subsequent promotion of cell death and suppression of tumor growth [19]. In a lung cancer model, TRPV4 activation normalizes tumor vasculature and promotes the effectiveness of chemotherapy [28]. However, these studies show that even very modest pharmacological activation of TRPV4 can promote EMT and breast cancer cell migration in the same model. Hence, dosing regimens may be critical for calcium-induced cell death in cancers that overexpress TRPV4, with maintained high levels of activator and/or combination with other agents required. Our results also provide evidence that TRPV4 inhibition may have anti-metastatic effects through effects on cancer cell migration [18] and also through reducing metastatic initiation via effects on EMT. The known role of EMT in the development of therapeutic resistance [5] provides another dimension to TRPV4 inhibition for the treatment of some breast cancers, the feasibility of which is demonstrated by the clinical trials of TRPV4 inhibitors for heart failure [29]. Given the temperature sensitivity of TRPV4 [30], future studies should assess the temperature effects of some of our identified phenotypes and effects, which may require the use of genetically encoded Ca^2+^ indicators, which are less prone to sequestration into intracellular organelles.

## 4. Materials and Methods

### 4.1. Cell Culture

The MDA-MB-468 human breast cancer cell line was obtained from The Brisbane Breast Bank, UQCCR, Brisbane, Australia, and maintained in Dulbecco’s Modified Eagle’s Medium (DMEM) with high glucose (D6546; Sigma-Aldrich, St Louis, MO, USA), supplemented with 10% fetal bovine serum (FBS) and 4 mM L-glutamine (25030; Thermo Fisher Scientific, Waltham, MA, USA). PMC42LA human breast cancer cell line was obtained from Dr Leigh Auckland, Deakin University, Melbourne, Australia [31,32], and maintained in Roswell Park Memorial Institute (RPMI)-1640 medium (11875085; Life Technologies, Carlsbad, CA, USA) with 10% FBS. Cells were maintained at 37 °C and 5% CO_2_ in a humidified incubator. For hypoxia experiments, cells were placed in a hypoxic incubator (37 °C, 1% O_2_, 5% CO_2,_ and 94% N_2_) for durations stated in the results. Cells were examined for mycoplasma every six months using MycoAlert kit (Lonza, Basel, Switzerland) and validated by short tandem repeat (STR) profiling using The GenePrint^®^ 10 System (Promega, Madison, WA, USA) at the Queensland Institute of Medical Research (QIMR) Berghofer Medical Research Institute, Brisbane, Australia.

### 4.2. Quantitative Real-Time RT–PCR

RNA isolation, purification, and synthesis of cDNA were performed as previously described [12]. Quantitative PCR was performed using TaqMan Gene Expression Assays and Fast Universal PCR Master Mix (4352042; Life Technologies, Carlsbad, CA, USA) in a StepOnePlus^TM^ Real-Time PCR instrument (Life Technologies). The following specific TaqMan Gene Expression Assays were used: vimentin (Hs00185584_m1), N-cadherin (Hs00983062_m1), AXL (Hs01064444_m1), SERPINE1 (Hs01126606_m1), Twist (Hs00361186_m1), Snail1 (Hs00195591_m1), CD44 (Hs01075861_m1), and CD24 (Hs02379687_s1). Comparative C_T_ (ΔΔC_T_) method and normalization to the ribosomal 18S sRNA (Gene Expression Assay 4319413E) were used to calculate relative target mRNA levels.

### 4.3. Immunoblotting

Protein isolation, immunoblotting, and densitometric analysis were performed as previously described [9]. Primary antibodies included vimentin (V6389, 1:750 dilution, Sigma-Aldrich), E-cadherin (14472, 1:1000 dilution, Cell Signaling Technology, Danvers, MA, USA), TRPV4 (ab39260, 1:500 dilution, Abcam, Melbourne, VIC, Australia), and β-actin (A5441, 1:10,000 dilution, Sigma-Aldrich). Secondary antibodies included goat anti-rabbit (170-6515, 1:10,000 dilution, Bio-Rad, Hercules, CA, USA) and goat anti-mouse (170-6516, 1:10,000 dilution, Bio-Rad) horseradish peroxidase conjugate. Vimentin, E-cadherin, and TRPV4 antibodies were incubated with membranes overnight at 4 °C in 5% non-fat milk. β-Actin and secondary antibodies were incubated for 1 h at room temperature.

### 4.4. siRNA Silencing

Dharmacon ON-TARGETplus SMARTpool siRNAs (a mixture of four rationally designed siRNAs; Millennium Science, Mulgrave, VIC, Australia) and DharmaFECT4 Transfection Reagent (0.1 µL per well) were used to transfect non-targeting siRNA (siNT; D-001810-10) or TRPV4 siRNAs (siTRPV4; L-004195-00) into the MDA-MB-468 cells. Cells were seeded at a density of 1 × 10^4^ (for EGF or GSK1016790A experiments) or 6 × 10^3^ (for 48 h hypoxia exposure) in 96-well plates and were transfected with siRNAs 24 h post-seeding.

### 4.5. Cell Migration Assay

Collagen matrices and live-cell imaging were used to assess cell motility. Ninety-six-well plates were coated with 50 µM collagen mixture comprising of 10× phosphate-buffered saline (8% volume/volume), DMEM (24% volume/volume), and collagen type I from bovine skin (C4243, Sigma-Aldrich) at a final concentration of 2 mg/mL), adjusted to physiological pH with 1 M NaOH. Plates were incubated at 37 °C, 5% CO_2_ for 1 h to form collagen gels. MDA-MB-468 cells were then seeded on top of the collagen gel at a density of 1000 cells per well. siRNA-mediated silencing of TRPV4 was conducted 24 h post-seeding as described above. Post silencing (24 h), cells were serum reduced (0.5% FBS) for 48 h and positioned on the stage of a JuLi^TM^ Stage Live Cell Imaging System (NanoEnTek Inc., Seoul, Korea) in a 37 °C hypoxia incubator. Starting from 72 h post exposure to hypoxia, bright field images (4× magnification) were captured from the center of wells every 15 min for a period of 12 h. The Manual Tracking plug-in of the ImageJ 1.49q software (NIH, Bethesda, MD, USA, https://imagej.nih.gov/ij/) was used to track single cells. Calculation and illustration of single cell movement over the 12 h period, were conducted using the Chemotaxis and Migration Tool V2.0 (Ibidi, Munich, Germany).

### 4.6. Measurement of Cytosolic Free Ca^2+^

[Ca^2+^]_CYT_ was measured in 96-well black CellBIND plates (CLS3340; Corning, Corning, NY, USA) using a fluorometric Imaging Plate Reader (FLIPR^TETRA^, Molecular Devices, San Jose, CA, USA) and a PBX no-wash Ca^2+^ Assay Kit (640175, BD Biosciences, San Jose, CA, USA) as previously described [33]. Calcium measurements were performed at room temperature. Data analysis was conducted using the ScreenWorks Software (v2.0.0.27, Molecular Devices). The response over baseline was measured as a relative measure of [Ca^2+^]_CYT_. Relative maximum peak and relative [Ca^2+^]_CYT_ at 800 s were calculated.

### 4.7. Patient Survival Analysis

Stratification of patient survival by TRPV4 and Red Module expression was done using the online survival analysis tool Kaplan-Meier Plotter [34]. Patients were stratified based on the TRPV4 probe 219516_at or the mean expression of the top 30 genes from the Red Module (Table S1), “Auto select best cutoff”, relapse-free survival (RFS), and lymph node status in women with Basal breast tumors (ER- and HER2-negative samples). Gene expression levels were determined by Kaplan-Meier Plotter and displayed as “low” (black line) or “high” (red line). The significance of stratification was determined by log-rank *p*-value and hazard ratios (HR) with 95% confidence intervals.

### 4.8. Gene Correlation Analysis

RNA-Seq data for breast cancer molecular markers, TRPV channels, and genes with the weighted gene co-expression network analysis (WGCNA) correlated network were sourced from the Cancer Genome Atlas (TCGA) [35] network via cBioPotal for Cancer Genomics [36,37]. In total, data for 1044 patients were analyzed, which includes 117 Basal-like (Basal), 59 HER2-Enriched (HER2), 361 Luminal A (LumA), 169 Luminal B (LumB), 113 Normal-like (N-Like), and 225 not yet classified tumors. RNA-Seq data were downloaded at RNA-seq by expectation maximization (RSEM [38]) and transformed via log2 mean-centering of expression for each gene. Normalized expression values were then clustered via Manhattan-based average-linkage clustering in Multiple Experiment Viewer (MeV [39]).

### 4.9. Weighted Correlation Network Analysis

Gene expression data for breast tumors were accessed from The Cancer Genome Atlas (TCGA) Project [35]; a total of 845 samples were analyzed, which included samples from all major breast tumor molecular subgroupings. Expression was analyzed using Weighted Co-expression Gene Network Analysis (WGCNA) in the R environment, as previously published [19]. In brief, WGCNA of breast tumor gene mRNA expression identified a co-regulated gene cluster, which included TRPV family genes, and this cluster was referenced by a color identifier as “Red Module”. Using measures of interconnectedness within and between modules, the 100 topmost interconnected genes of the Red Module were identified (Table S1). Gene ontology analysis was performed using the AmiGO [40] bioinformatics tool (http://amigo.geneontology.org/amigo), with Bonferroni multiple-comparison correction.

### 4.10. Weighted Correlation Network Analysis

GraphPad Prism (La Jolla, CA, USA, www.graphpad.com) was used for statistical analysis. Specific statistical tests and significance for each experiment are stated in corresponding figure legends.

## 5. Conclusions

In summary, TRPV4 is a receptor-selective regulator of activated calcium influx in breast cancer cells that overexpress TRPV4 and pharmacological activation of TRPV4 is sufficient to induce EMT in these cells. Our studies highlight new roles for TRPV4 in breast cancer and identify that some ion channels are not just regulators of specific elements of EMT but can be the driver of this phenomenon.

## Supplementary Materials

Supplementary materials can be found at https://www.mdpi.com/1422-0067/21/24/9417/s1, Table S1: Top 100 most interconnected genes of the Red Module; Figure S1: Confirmation of TRPV4 siRNA-mediated silencing; Figure S2: Comparison of TRPV4 mRNA levels in MDA-MB-468 and PMC42LA cells.

## Abbreviations

Basal	Basal-like
DMEM	Dulbecco’s Modified Eagle’s Medium
EGF	epidermal growth factor
EMT	epithelial to mesenchymal transition
E-Cad	E-cadherin
FBS	fetal bovine serum
FLIPR	fluorometric imaging plate reader
HER2	HER2-Enriched
LumA	Luminal A
LumB	Luminal B
MeV	Multiple Experiment Viewer
N-Like	Normal-like
PAR2	protease-activated receptor 2
RFS	relapse-free survival
RSEM	RNA-seq by expectation maximization
RPMI	Roswell Park Memorial Institute
TCGA	the Cancer Genome Atlas
TGFβ1	transforming growth factor β1
WGCNA	weighted gene co-expression network analysis
