# Supplementary material for: Activation of the Ion Channel TRPV4 Induces Epithelial to Mesenchymal Transition in Breast Cancer Cells
Source: Int J Mol Sci. 2020 Dec 10;21(24):9417. doi: 10.3390/ijms21249417 (PMC7764818; doi:10.3390/ijms21249417)
Supplement: Supplementary file 1 [file ijms-21-09417-s001.pdf]

Activation of the ion channel TRPV4 induces epithelial to mesenchymal transition in breast cancer cells

Iman Azimi, Mélanie Robitaille, Kaela Armitage, Choon Leng So, Michael Milevskiy, Korinne Northwood, Huai Fang Lim, Erik W Thompson, Sarah Roberts-Thomson, Gregory Monteith

Supplemental material

Table S1. Top 100 most interconnected genes of the Red Module.

Gene	kTotal	kWithin	kOut	kDiff
ROPN1	2.665705	2.422587	0.243117	2.17947
FOXC1	3.021773	2.154989	0.866784	1.288205
ANXA8	1.986219	1.976218	0.010001	1.966217
CCDC82	2.621327	1.87143	0.749897	1.121534
ANXA8L2	1.73774	1.726229	0.011511	1.714718
KRT5	1.705635	1.659191	0.046444	1.612747
MAML2	2.101074	1.643174	0.4579	1.185274
KRT6B	1.643053	1.636257	0.006796	1.629461
KRT17	1.641288	1.612715	0.028573	1.584142
DSC3	1.578761	1.543334	0.035427	1.507906
SFRP1	1.672026	1.534475	0.137551	1.396923
FAM126A	2.247748	1.530447	0.717301	0.813146
PLEKHG1	2.134668	1.4686	0.666068	0.802532
FAM171A1	1.735645	1.395844	0.339801	1.056043
SFRS13B	2.068389	1.352625	0.715764	0.636861
ROPN1B	1.417966	1.337803	0.080163	1.257639
HAPLN3	2.567416	1.328238	1.239178	0.08906
KRT14	1.404378	1.314709	0.08967	1.225039
MRAS	1.611315	1.290898	0.320417	0.970481
KLK7	1.284678	1.274158	0.01052	1.263638
VGLL1	1.902127	1.264776	0.637351	0.627425
RGMA	1.463472	1.25475	0.208721	1.046029
STAC	1.694877	1.232201	0.462675	0.769526
MIA	1.266602	1.232099	0.034503	1.197596
GABRP	1.281528	1.223896	0.057632	1.166264
KLK6	1.179502	1.164228	0.015274	1.148954

BCL11A	1.46638	1.1067	0.35968	0.74702
EGFR	1.334853	1.04141	0.293443	0.747967
B3GNT5	1.76232	1.032807	0.729513	0.303294
SOX10	1.105534	1.026404	0.07913	0.947273
CHST3	1.267812	1.01297	0.254842	0.758127
ACSL4	2.248714	1.00454	1.244174	-0.23963
DSG3	0.999424	0.989233	0.010191	0.979043
PPP1R14C	1.27081	0.959646	0.311164	0.648483
DZIP1	1.682704	0.930192	0.752512	0.17768
FAT2	1.082889	0.929626	0.153263	0.776363
ANXA1	1.374404	0.911523	0.462882	0.448641
KLK5	0.894596	0.883393	0.011203	0.872191
FAM49A	1.69676	0.820864	0.875897	-0.05503
TRIM29	0.825988	0.804831	0.021157	0.783674
MFGE8	0.983043	0.789784	0.193259	0.596525
MSN	1.650027	0.784187	0.86584	-0.08165
CLCN4	1.155642	0.7801	0.375542	0.404559
GLIPR2	1.573334	0.766479	0.806855	-0.04038
ART3	1.893752	0.750881	1.142871	-0.39199
KLK8	0.745361	0.733792	0.011569	0.722224
PLAGL1	1.12737	0.72682	0.400549	0.326271
MID1	0.923478	0.710744	0.212733	0.498011
MOBKL2B	0.858304	0.703438	0.154866	0.548572
KRT16	0.765016	0.695234	0.069782	0.625452
OCA2	1.009394	0.676128	0.333266	0.342862
FMNL2	1.458647	0.672312	0.786335	-0.11402
PTX3	0.770324	0.66488	0.105444	0.559436
KRT6C	0.676489	0.664285	0.012204	0.652081
PRNP	0.802234	0.65275	0.149484	0.503266
MAP7D3	0.986342	0.608434	0.377907	0.230527
CNTNAP3	0.687036	0.600736	0.0863	0.514437
CRYAB	0.672368	0.592552	0.079815	0.512737
ARL4C	1.268734	0.584872	0.683861	-0.09899
ST8SIA1	0.919932	0.58117	0.338762	0.242408
RBMS1	0.895559	0.570014	0.325545	0.244468
QKI	0.992983	0.564889	0.428093	0.136796
SAA2	0.596563	0.56342	0.033143	0.530276
CSDA	0.633992	0.563419	0.070573	0.492847

BBOX1	0.598402	0.562301	0.036101	0.5262
EPHB1	0.623708	0.553694	0.070014	0.48368
FERMT1	0.569548	0.538114	0.031434	0.50668
SAA1	0.606084	0.53795	0.068134	0.469816
GPM6B	0.598787	0.536228	0.062559	0.473669
CSDAP1	0.585596	0.534531	0.051064	0.483467
SPRR2A	0.534671	0.533892	0.000779	0.533113
ZNF454	0.641807	0.528185	0.113622	0.414563
TTYH1	0.567384	0.52632	0.041064	0.485256
C9orf170	0.792361	0.515967	0.276394	0.239574
RASGEF1C	0.678879	0.514674	0.164204	0.35047
SPRR2D	0.512369	0.512051	0.000318	0.511733
DMD	0.694321	0.503953	0.190368	0.313585
IL34	0.645401	0.487783	0.157618	0.330165
PLA2G4A	0.640522	0.482759	0.157763	0.324997
LEMD1	0.816112	0.481845	0.334267	0.147577
UGT8	0.598597	0.463255	0.135341	0.327914
WWTR1	0.64414	0.461808	0.182332	0.279475
BOC	0.627433	0.459281	0.168152	0.291129
ROR1	1.033942	0.457598	0.576344	-0.11875
FSCN1	0.889122	0.448502	0.44062	0.007882
SIRPA	1.107469	0.447571	0.659898	-0.21233
SOX8	0.499786	0.444639	0.055147	0.389493
CHST2	1.087465	0.436692	0.650773	-0.21408
AGPAT4	0.782968	0.436565	0.346403	0.090162
SERPINB5	0.441897	0.43599	0.005908	0.430082
MPP6	0.736232	0.428021	0.308211	0.11981
CARD6	1.348641	0.426075	0.922566	-0.49649
DLX6	0.463047	0.425212	0.037835	0.387377
CHODL	0.835284	0.423291	0.411993	0.011298
FZD9	1.009899	0.418499	0.5914	-0.1729
PTCHD1	0.525988	0.4175	0.108487	0.309013
PSAT1	1.352388	0.405541	0.946848	-0.54131
C17orf51	0.711313	0.404848	0.306465	0.098383
TBX19	0.713067	0.402841	0.310225	0.092616
KRT6A	0.406454	0.400453	0.006001	0.394452

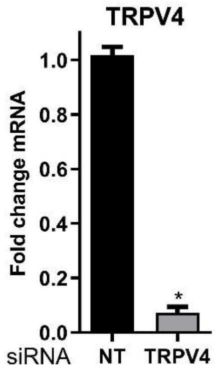

Figure S1. Confirmation of TRPV4 siRNA-mediated silencing. * $p < 0.0001$, unpaired t-test. n = 3, mean ± SD.

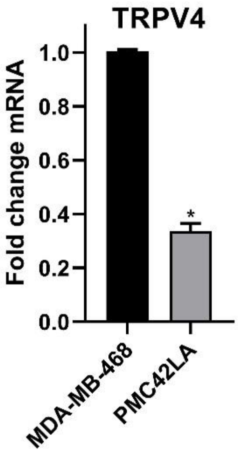

Figure S2. Comparison of TRPV4 mRNA levels in MDA-MB-468 and PMC42LA cells. * $p < 0.0001$, unpaired t-test. n = 3, mean ± SD.
